# High Pressure Pneumatic Forming of Ti-3Al-2.5V Titanium Tubes in a Square Cross-Sectional Die

**DOI:** 10.3390/ma7085992

**Published:** 2014-08-20

**Authors:** Gang Liu, Jianlong Wang, Kexin Dang, Zejun Tang

**Affiliations:** 1 College of Materials Science and Engineering, Harbin Institute of Technology, Harbin 150001, China; E-Mails: wangjianlonghit@gmail.com (J.W.); w15045852476@gmail.com (K.D.); 2 National Key Laboratory for Precision Hot Processing of Metals, Harbin Institute of Technology, Harbin 150001, China; 3 Mechanical and Electrical College, Nanjing University of Aeronautics and Astronautics, Nanjing 210016, China; E-Mail: zjtang@nuaa.edu.cn

**Keywords:** high pressure pneumatic forming (HPPF), corner filling, strain rate sensitivity, electron back scattering diffraction (EBSD)

## Abstract

A new high strain rate forming process for titanium alloys is presented and named High Pressure Pneumatic Forming (HPPF), which might be applicable to form certain tubular components with irregular cross sections with high efficiency, both with respect to energy cost and time consumption. HPPF experiments were performed on Ti-3Al-2.5V titanium alloy tubes using a square cross-sectional die with a small corner radius. The effects of forming of pressure and temperature on the corner filling were investigated and the thickness distributions after the HPPF processes at various pressure levels are discussed. At the same time, the stress state, strain and strain rate distribution during the HPPF process were numerically analyzed by the finite element method. Microstructure evolution of the formed tubes was also analyzed by using electron back scattering diffraction (EBSD). Because of different stress states, the strain and strain rate are very different at different areas of the tube during the corner filling process, and consequently the microstructure of the formed component is affected to some degree. The results verified that HPPF is a potential technology to form titanium tubular components with complicated geometrical features with high efficiency.

## 1. Introduction

In comparison with liquid-based forming operations, in the area of forming at elevated temperatures, gas offers the chance to provide higher temperatures due to its high temperature resistance in contrast to most liquids. Since gases have low specific heat capacities as compared to water, cold gas can be guided into the heated hollow body without causing significant cooling of the forming zone [1]. Pre-heating of the gas is therefore not needed. There are many kinds of forming processes on pneumatic forming at elevated temperatures, e.g., super-plastic forming (SPF), quick-plastic forming (QPF), hot metal gas forming (HMGF). Except for the SPF process, the other processes are characterized by short cycle times ranging from a few seconds to minutes. Among the studies of the QPF process, Jarrar *et al.* [2] found that a common failure mode during QPF of non-ferrous materials in plane strain was splitting (or rupture) in the vicinity of the die entry radius and major contributing factors were sheet-die friction and die geometry. Krajewski *et al.* [3] also studied the effect of friction on necking around the entry corner of the die and found that with a low-friction condition necking was observed below the entry corner of the die. Boissiere *et al.* [4] discussed some similarities and differences between QPF and SPF and pointed out that the deformation mechanism for QPF was viscous glide controlling dislocation creep and the deformation mechanism for SPF was grain boundary sliding. Wu *et al.* [5] performed tensile tests and shallow pan rapid gas blow forming to explore the deformation behavior of AZ31B Mg alloy sheet and found that the fillet radius of the rectangular pan to be one of the key factors influencing forming time on closed die gas blow forming. The HMGF process is mainly used to form tubular components. Liu and Wu [6] investigated the HMGF process of AZ31 magnesium alloy tubes and pointed out that the hybrid deformation mechanism of the material affected the final microstructure of the workpiece because of the non-uniform temperature caused by induction heating and non-uniform strain. Kim *et al.* [7] showed that an aluminum suspension component was successfully fabricated by HMGF at 793 K under a constant gas pressure of 7 MPa after a gas input time of 70 s. They also analyzed the forming and fracture behavior of aluminum alloy tube with the aid of finite element (FE) simulation, into which the Zener-Hollomon parameter based failure criterion was incorporated. The simulation results could explain the experimental results and showed that the actual forming mode during HMGF was close to uniaxial or biaxial tension mode.

Compared with SPF, QPF and HMGF can shorten the cycle time greatly due to the higher strain rate (usually between 0.001–0.01 s^−1^). Aluminum and magnesium alloys are mainly used in the two pneumatic forming processes and the forming pressure is usually lower than 10 MPa [2,3,4,5,6,7]. For titanium components, SPF is mainly used to form them. This process is an effective process to produce components with complex geometries and large deformation, but the cycle times vary from 30 min to 2 h for SPF due to its lower strain rate (usually lower than 0.001 s^−1^) [8]. In conclusion, the QPF or HMGF process at higher pressure can be spread to some titanium components with a small expansion ratio (usually smaller than 50%). So, to realize fast forming of titanium tubular components with a small expansion ratio but with obvious local features (such as small corner radii), a new pneumatic forming process was developed based on QPF, HMGF, and hydroforming. This process can be called high pressure pneumatic forming (HPPF) and has two obvious peculiarities: (a) The pressure ranging from 10 to 70 MPa is supplied to obtain higher strain rate (usually above 0.01 s^−1^) and the forming time is within a few seconds; (b) The die can be overall heated to 800 °C rapidly (usually within 1 h) using inductive heating. The induction heating system uses a high frequency induction coil in order to concentrate the energy into the die and the energy cost is much less. Compared with the hydroforming process, in the HPPF process, the forming pressure decreases with the decreasing yielding stress, and the springback weakens, obviously because of the smaller residual stress due to the elevated temperature. For titanium alloys, HPPF can achieve the required elongations and guarantee the small radii of the tubular components more easily than hydroforming. Neugebauer and Schieck [9] developed a device with a maximum pressure of 80 MPa. The device was mainly used to form high strength steel tubes. During this forming process, the preheated tube was formed at high pressure in the cold die and then cooled quickly. Instead of the uniaxial tensile test, Elsenheimer and Groche [10] set up hot tube bulge test equipment to obtain stress-strain curves of the tube at elevated temperatures through this test. In this equipment, the tube is heated by an induction system and the gas pressure can reach 35 MPa. Drossel and Pierschel [11] developed a measuring instrument to determine the temperature variation curve of the gas in the tube with a measuring frequency of at least 1 Hz during high pressure pneumatic forming of high strength steel. They confirmed that it was possible to determine the temperature variation curve of the gas and the maximum temperature of the active media was up to 500 °C. He [12] measured the formability of TA2 pure titanium at different temperatures and determined the best forming parameters, the results showed that a good formability could be attained at the proper temperature, at which the uniform elongation has the biggest value, but not the total elongation with necking. Liu [13] set up an HPPF device with a maximum pressure of 70 MPa, and conducted forming experiments of a TA15 Ti-alloy component with an irregular cross section, at temperatures of 800–850 °C.

In the hydroforming of square section components at room temperature, obvious stress concentration occurs at the transition zone between the straight wall area and the corner area, and the forming pressure required for the transition zone is larger than that for other areas [14]. Experimental results also demonstrated convincingly that there was a limit for the transition corner radius at a certain pressure level during hydroforming at room temperature [15]. However, few investigations have focused on corner filling behavior at elevated temperature, especially for the HPPF process for titanium tubes. In the present work, HPPF experiments were performed on Ti-3Al-2.5V titanium alloy tubes using a round-to-square die with a small corner radius. The effects of forming pressure on the circumferential thickness distribution were investigated and the corner filling behavior under various temperature and pressure levels was discussed. The stress state, strain distribution and strain rate distribution of the component during forming were quantitatively analyzed using DEFORM-2D software. Finally, the microstructure evolution of typical regions at the cross section of the tubes formed at 850 °C was investigated by using electron back scattering diffraction (EBSD).

## 2. Experimental Procedure and Finite Element Model

### 2.1. Material and Experimental Setup

**Figure 1:**
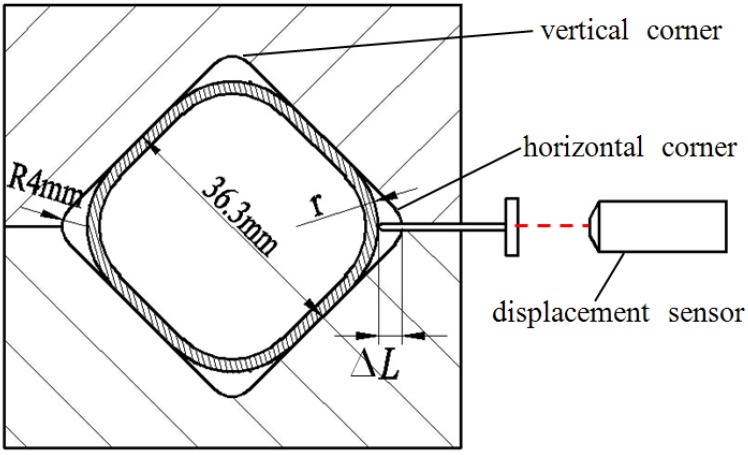
Round-to-square die set.

Ti-3Al-2.5V titanium alloy tubes of 40 mm in outer diameter, 200 mm in length and 2.1 mm in thickness, were used in the experiments. The analyzed chemical compositions are given in Table 1. Figure 1 illustrates the round-to-square die set that was designed and manufactured for the HPPF experiments. The maximum expansion ratio of the tube during bulging was 10%. Because the distance between the two opposite faces of the designed part was less than the original tube diameter, the diagonal parting die set was adopted such that the preforming process would take place during the closing of the die. An HPPF device with maximum pressure of 70 MPa developed by the Harbin Institute of Technology was used to bulge the tubes inside the closed die in order to fill the die cavity. Initially, the die was heated overall to the forming temperature rapidly using inductive heating. Then the tube was put into the die and heated to the forming temperature through heat conduction with the die. After the temperature of the tube became stable, the pressurized gas was used to expand the tube inside the closed die.

**Table 1:** Component analysis results of TA18 titanium alloy (mass fraction, %).

TA18 (Ti-3Al-2.5V)							
Al	V	O	Fe	C	N	H	Ti
2.85	2.36	0.054	0.037	0.016	0.012	0.0014	Bal.

In order to explore the effect of lubrication on the preforming process during the closing of the die, the test tube was heated up and stabilized at 850 °C in the die for 3 min, and then the die was closed. After the preforming process, it was immediately removed from the die without being expanded. HPPF experiments were carried out at various temperatures and pressure levels. Boron nitride was chosen as the lubricant during the preforming process and HPPF experiments. In order to investigate the effects of the forming pressure on the corner filling behavior and thickness distribution, four different constant pressure levels were used in the HPPF experiments at 850 °C: 15, 20, 25 and 30 MPa. HPPF experiments were also conducted at various temperatures to explore the corner filling behavior preliminarily. At a pressure level of 30 MPa, the corner was formed respectively at a temperature of 700, 750, 800, 850 °C and the forming time was limited to 300 s. During the HPPF experiments, A KEYENCE laser displacement sensor was used to measure the displacement of the horizontal corner in real time, as shown in Figure 1. The data recorded was used to calculate the horizontal corner radius in real time. During the corner filling process, the instantaneous radius and displacement of the corner can be expressed as Equation (1):

*r* = *R* + 0.414*∆i* (1)

where *r* is the instantaneous radius of the corner; *R* is the radius of the die; *∆i* is the instantaneous displacement of the corner [16].

### 2.2. Finite Element Model

**Figure 2:**
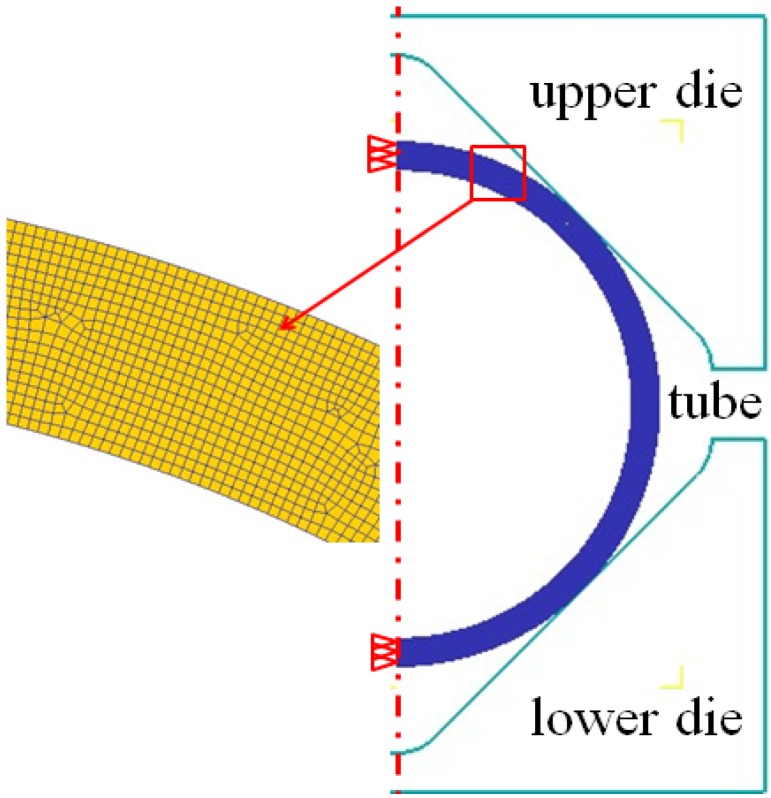
Finite element model of the square cross-section.

The goals of this study are to investigate the strain rate distribution, strain distribution and stress states of Ti-3Al-2.5V tubes in the round-to-square HPPF process. DEFORM 2D10.2 which is based on the flow formulation approach using an updated Lagrange procedure was used to simulate the HPPF process. As shown in Figure 2, one half of a plane strain model was set up according to the symmetrical profile. The corner filling process at 850 °C and 30 MPa was simulated. During the simulation, the die was assumed to be rigid, the tube was plastic, and four-node isoparametric elements were used. Through experimental measurement and primary simulation, it is known that the maximum strain rate during the HPPF of this workpiece is lower than 0.1 s^−1^. On the other hand, as in the case of HPPF, the strain rate is mainly kept larger than 0.001 s^−1^. Therefore, the flow curves obtained from tensile tests at 850 °C and three strain rates (0.1, 0.01, 0.001 s^−1^) were used as the material model. During simulation, the flow stress for the strain rate between 0.001 and 0.1 s^−1^ can be linearly interpolated using a weighted method based on the three flow curves [17].

### 2.3. Microstructure Investigation

The microstructures of the as-received and formed tubes were characterized with the electron back scattered diffraction (EBSD) method on a scanning electron microscope (SEM, Quanta 200 FEG-SEM, FEI company, Hillsboro, OR, USA) equipped with an EBSD detector and analysis software TSL OIM 6.14. The specimens for EBSD were prepared by electro-chemical polishing. In the present study, the EBSD results including grain size figures were analyzed. The grain size figure is a color coded figure where the color gives an indication of grain size. Different colors represent different size ranges of the grains.

## 3. Results and Discussion

### 3.1. Effect of Lubrication on Preforming Process while Closing the Die

**Figure 3:**
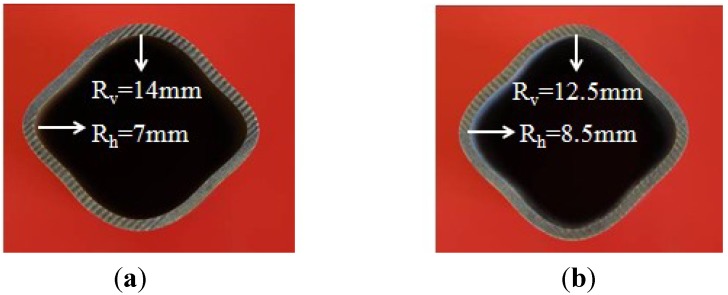
(**a**) The preformed cross section of the tube without lubrication; (**b**) the preformed cross section of the tube with BN lubrication.

While closing the die, the tube was preformed to the similar shape of the tool cavity, only with the corner areas untouched. Figure 3 shows the preformed cross section of the tubes with and without lubrication. After the preforming process, at the horizontal corner areas a bending deformation larger than the one at the vertical corner areas was noticed. When the tube was preformed without lubrication, the surface friction force caused by high temperature restricted the relative sliding between the die and the tube. As a result, bending deformation concentrated at the horizontal corner areas while the upper die moved down and the horizontal corner radius was only 7 mm compared with the vertical corner radius of 14 mm, as shown in Figure 3a. But when the tube was preformed with lubrication, it was beneficial to the relative sliding between the die and tube due to the decreased surface friction force. As shown in Figure 3b, the horizontal corner radius is 8.5 mm compared with the vertical corner radius of 12.5 mm. The horizontal corner radius is closer to the vertical corner radius after being preformed with lubrication and it is beneficial to decrease the thickness variation after the subsequent expanding process.

### 3.2. Effects of Pressure Levels on the Corner Filling Process and Thickness Distribution

**Figure 4:**
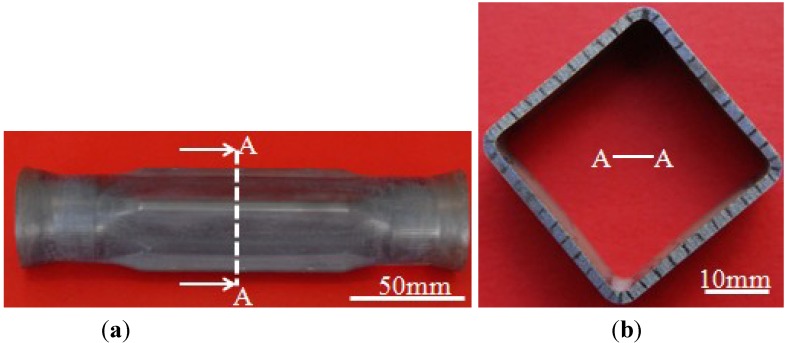
(**a**) Fully formed component with outer radius of 4 mm; (**b**) middle cross-section of the fully formed component.

**Figure 5:**
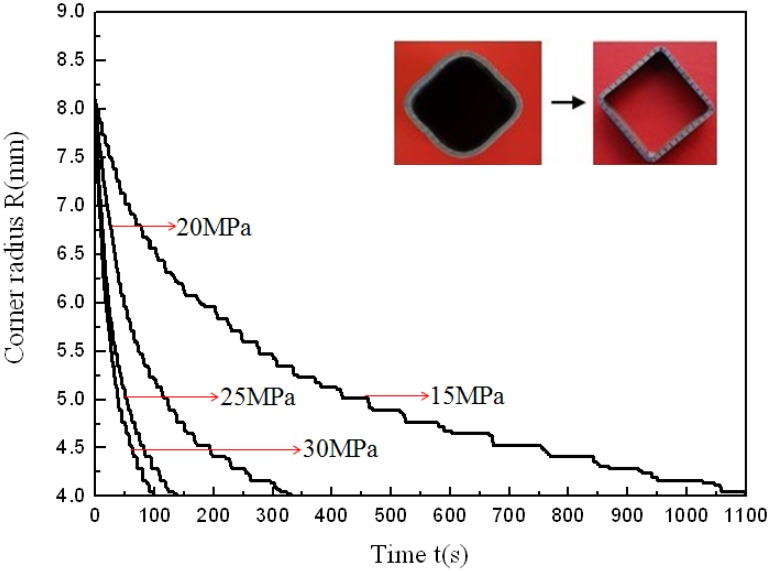
Corner filling process at 850 °C and four different pressure levels.

**Figure 6:**
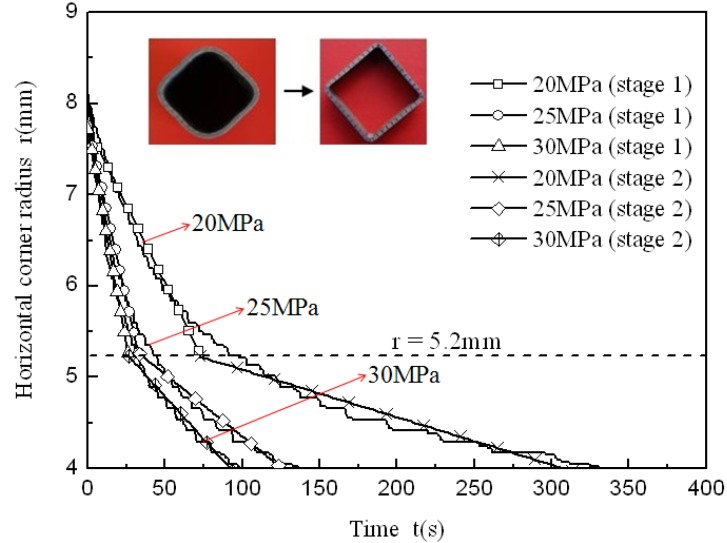
Corner filling curves at three different pressure levels which are linearly fitted.

After forming at 850 °C and using four different pressure levels, tubular components with an outer radius of 4 mm were all fully formed and one of the fully formed components is shown in Figure 4. The corner filling process is shown in Figure 5. During the preforming process, the corner areas of the tube do not contact with the die so that the preforming corner radii are not controlled precisely. Therefore, there is a slight difference in the corner radius of the preformed tubes. Besides, 8 mm is the value at which the four corner filling curves all start to change steadily. So the data of radii from 8 and 4 mm are analyzed to reduce errors caused by the preforming process and measuring. Because of the strain rate sensitivity at elevated temperature, the corner radii of the forming tubes depend on the internal pressure levels and forming time. This is different from corner filling at room temperature in which the corner radii of the forming tube depend only on the internal pressure levels. As is well known, forming a smaller corner needs a higher pressure in the hydroforming process. At room temperature and with a constant pressure level, as the radius reaches a certain value, the corner filling process stops because of the work hardening effect and the smaller radius. At elevated temperature and with a constant pressure level, as the radius gets smaller and smaller, the corner filling does not stop although it becomes slower and slower. This results from the strain rate sensitivity at elevated temperature. When the strain rate decreases, the flow stress also decreases to permit continuous deformation. Figure 5 shows that the same corner radius could be achieved with different pressure levels at the identical forming temperature, indicating that pressure levels might not be one of the major factors influencing formability but can notably affect the forming time. The corner radius changing from 8 to 4 mm needs 100 s at a pressure level of 30 MPa, but needs 1100 s at a pressure level of 15 MPa. In order to better understand the effect of pressure levels on the corner filling rate, the corner filling curves at different pressure levels were divided into two stages, then the curves at stage 1 and stage 2 were linearly fitted where the absolute values of the slopes refer to the average filling rate. Through the variation trend of the corner filling curves, it is found that values between 5 and 5.5 mm divide the whole curve clearly into two stages. When 5.2 mm is the value at which the first stage ends and the second one begins, every stage of the corner filling curves at different pressure levels can be linearly fitted and can be used for comparing corner filling rates between two stages. Therefore, 5.2 mm is chosen as the point of division. Besides, the corner filling time at 15 MPa is too prolonged for the HPPF process and the corner filling curve for 15 MPa is not considered. So corner filling curves at 20, 25 and 30 MPa are chosen for the analysis, as shown in Figure 6. Table 2 shows the average filling rate of stage 1 and stage 2 at three different pressure levels, in which ∆r_1_ refers to the average filling rate of stage 1 and ∆r_2_ refers to the average filling rate of stage 2. It is found that ∆r_2_ is much smaller than ∆r_1_ at the same pressure level. As the pressure level is increased, both ∆r_1_ and ∆r_2_ increase while ∆r_1_/∆r_2_ decreases. This indicates that the higher pressure level can not only increase the average filling rate but also decreases the descent gradient of the filling rate.

**Table 2:** The average filling rate of stage 1 and stage 2 at different pressure levels.

Pressure (MPa)	∆r_1_ (mm·s^−1^)	∆r_2_ (mm·s^−1^)	∆r_1_/∆r_2_
20	0.034	0.005	6.80
25	0.076	0.014	5.43
30	0.094	0.019	4.95

**Figure 7:**
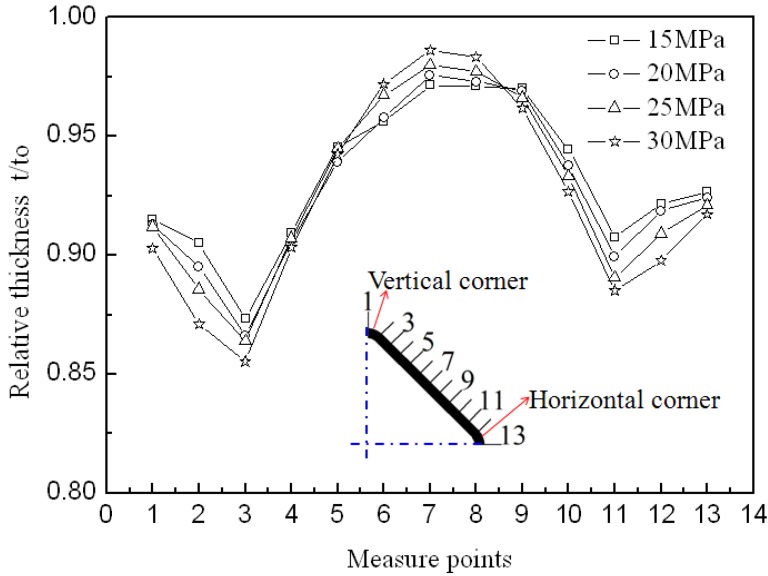
Thickness distribution at 850 °C and at different pressure levels.

Figure 7 illustrates the circumferential thickness distribution of tubular components at 850 °C and four different pressure levels. It can be seen that the minimum thickness occurs at the transition zone between the straight wall area and the vertical corner area and the maximum thickness occurs at the middle of the straight wall area. Because the horizontal corner radius is smaller than the vertical corner radius after the preforming process, the thickness from the horizontal corner to the middle of the straight wall area is slightly greater than that from the vertical corner to the middle of the straight wall area after the expanding process. The maximum thinning ratio does not significantly change and is 12.86%, 13.38%, 13.62%, 14.48% at the pressure levels of 15, 20, 25, 30 MPa, respectively. However, higher pressure induces a little more thinning at the transition zone between the straight wall area and the vertical corner area (Figure 7, point 3). This is because the smaller friction force at lower pressure makes the deformation in the straight wall area easier once the tube is in contact with the die. Besides, the strain rate sensitivity index is affected by the strain rate and it is related to the thinning behavior of the material. Li *et al.* [18] studied the effects of strain rate on the formability of Ti-6Al-4V alloy sheet and found that the strain rate sensitivity index decreased with increasing the strain rate. Salam [19] studied the flow stress-strain rate behavior of Ti-3Al-2.5V alloy and also found that the strain rate sensitivity index decreased with increasing the strain rate. According to these results, lower pressure induces smaller flow stress and lower strain rate in HPPF experiments, so the strain rate sensitivity index is probably larger and this can result in more uniform deformation. In the future, much more work will be done to verify this inference. The reason for the occurrance of the minimum thickness at the transition zone instead of at the middle part of the corner is probably because the stress states are different at different areas during the corner filling process. Through the HPPF process, the maximum thinning ratio of the cross section is smaller than 15%, which can meet the demand of most applications and correct lubrication improves the thickness uniformity.

### 3.3. Effect of Temperature on Corner Filling Process

**Figure 8:**
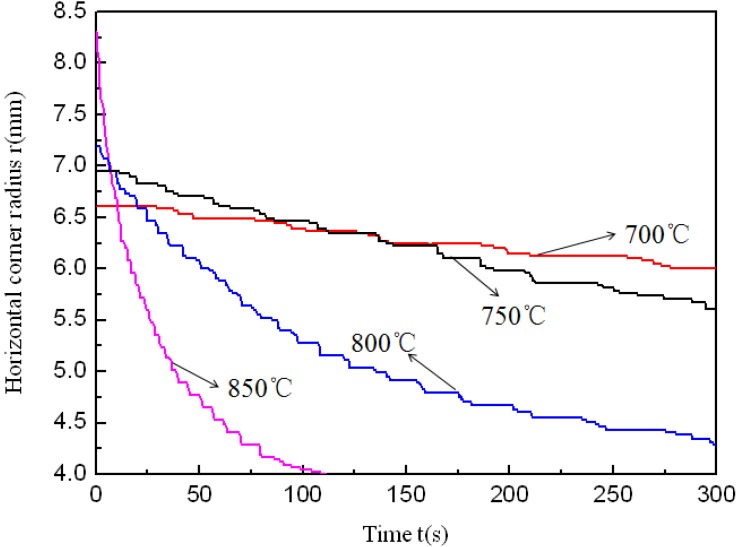
Corner filling process at various temperatures and at the same pressure level of 30 MPa.

Table 3 shows the corner radii of the components formed in 300 s. It can be seen that the corner radii are smaller at higher temperature and at a constant pressure level of 30 MPa. The corner filling process is shown in Figure 8. Although different temperatures result in a more obvious difference in the horizontal corner radius of the tubular components after the preforming process, it is still found that the filling rate is faster and the minimum corner radius is smaller at higher temperature with a constant pressure level of 30 MPa. The corner filling is easier because of decreased flow stress and weakened work hardening effect at higher temperature. In Figure 8, it is also found that the corner filling process does not stop at 700 °C and 30 MPa, although it becomes slower and slower. This indicates that the strain rate sensitivity has started to affect the corner filling at 700 °C. However, for the corner filling process at 700 °C, a constant pressure level of 30 MPa is very low and this induces low strain rate and small flow stress, so it is always slower than that at higher temperature and with the same pressure level.

**Table 3:** Corner radii at various temperatures in 300 s.

T (°C)	Time (s)	Pressure (MPa)	Vertical corner radius (mm)	Horizontal corner radius (mm)
700	300	30	8	6
750	300	30	7	5.5
800	300	30	5	4.5
850	300	30	4	4

### 3.4. Finite Element Method Analysis of Corner Filling Process

**Figure 9:**
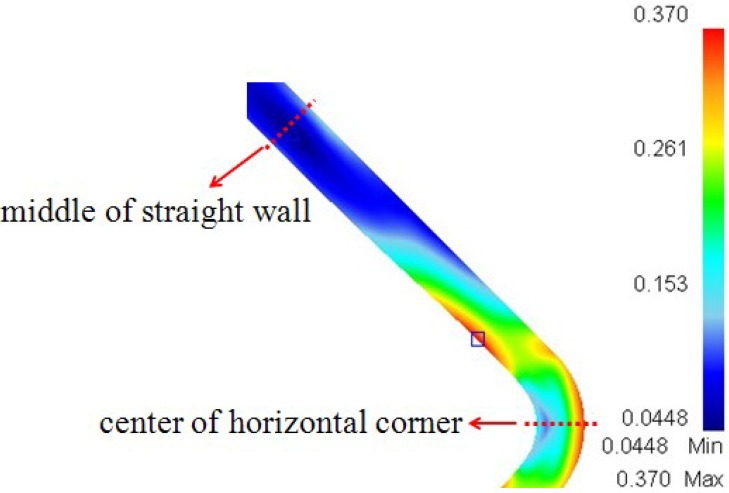
Effective strain distribution at the cross-section.

**Figure 10:**
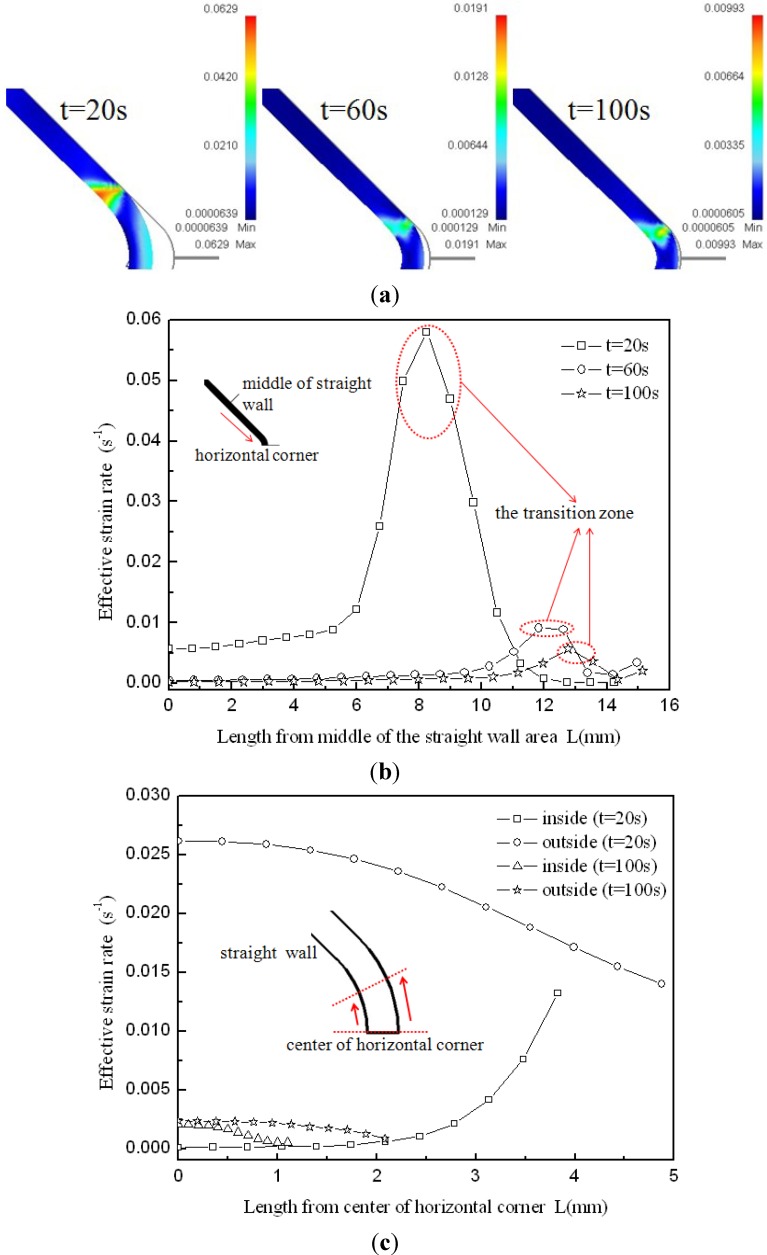
(**a**) Finite Element Method results of effective strain rate distribution; (**b**) values of effective strain rate at the inside of the component; (**c**) values of effective strain rate at the inside and outside of the horizontal corner.

At the end of the corner filling process, the effective strain distribution at the cross-section is shown in Figure 9. From the thickness distribution in Figure 7, it is found that the thickness difference between the vertical corner and horizontal corner is very small, therefore only the horizontal corner radius is measured during the HPPF process and analyzed in the following context. It can be seen that between the straight wall area and horizontal corner area the maximum effective strain of 0.37 is located at the inside of the transition zone, but the effective strain is different along the thickness direction, especially at the corner area. The effective strain just achieves 0.116 at the inside of the horizontal corner, while it achieves 0.330 at the outside of the horizontal corner. This is probably because of different stress states at the inside and outside of the corner. The effective strain rate distribution at different moments is given in Figure 10a, the values of effective strain rate from the middle of the straight wall area to the center of the horizontal corner area at the inside of the component are given in Figure 10b, and the values of the effective strain rate at the inside and outside of the horizontal corner are given in Figure 10c. As shown in Figure 10a,b, the maximum effective strain rate always occurs at the transition zone during the corner filling process and it nearly achieves 0.06 s^−1^ at the time of *t* = 20 s, but the effective strain rate at the straight wall area and corner area are much smaller. As a result, deformation at the transition zone is much larger than other areas. It is also found that the effective strain rate at the transition zone becomes slower clearly during the corner filling process and this is the main reason for the corner filling rate becoming slower at constant pressure levels. For the straight wall area, the effective strain rate is 0.0065 s^−1^ at the time of 20 s, but it is nearly zero at the moment of *t* = 60 s. This indicates that small deformation at the straight wall area mainly occurs at the beginning of corner filling. For the corner area, it can bulge freely during the corner filling process and the situation is much more complicated. As shown in Figure 10c, the effective strain rates at the inside and outside of the corner are very different at the time of *t* = 20 s: for the outside of the corner, the effective strain rate can be above 0.025 s^−1^ at the center of the corner and decreases to 0.014 s^−1^ gradually far away from the center; for the inside of the corner, the effective strain rate is nearly 0 at the center and increases to 0.014 s^−1^ gradually far away from the center. However, the effective strain rates at the inside and outside of the corner become very close at the time of 100 s and the effective strain rate at the center of the inside corner can achieve 0.002 s^−1^. This indicates that a small deformation around the center of the inside corner mainly occurs at the end of the corner filling. Besides, the effective strain rate at the outside of the corner is always higher than that at the inside of the corner during the corner filling process. Hwang and Chen [20] indicated that the tube at the transition zone was subjected simultaneously to a tensile stress and a compressive stress. Because of this, material at the transition zone flows more easily and this leads to a higher strain rate. However, the stress states at other areas are also very important with respect to their deformation during the corner filling process and need to be further discussed.

**Figure 11:**
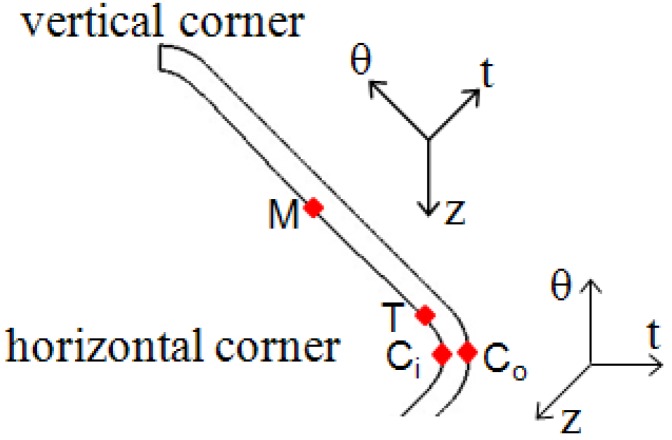
Position of typical feature points.

**Figure 12:**
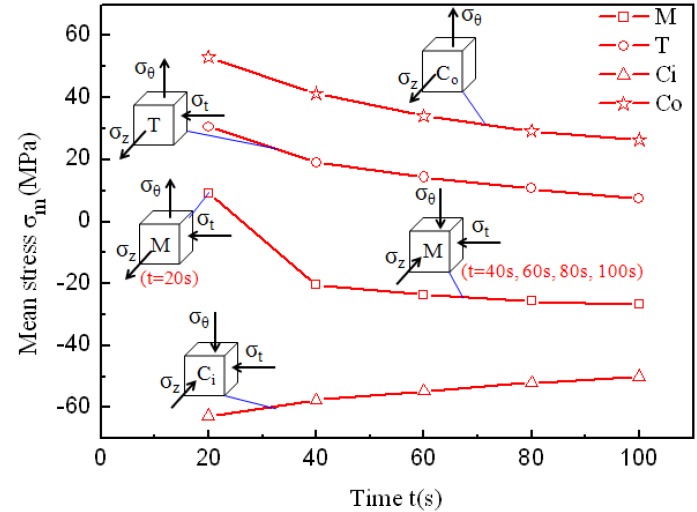
The stress states and mean stresses at the typical feature points during the corner filling process.

With the simulation results, the stress states of typical feature points during the corner filling process are obtained, including the middle point of the straight wall at the inside of the tube (Figure 11, point M), the transition point at the inside of the tube (Figure 11, point T), and the central points of the horizontal corner at the inside and outside of the tube (Figure 11, point C_i_ and point C_o_). The positions of these points are shown in Figure 11 and the stress states during corner filling process of these points are shown in Figure 12. For a tubular component, σ_θ_ refers to the stress in the hoop direction; σ_t_ refers to the stress in the thickness direction; and σ_z_ refers to the stress in the axis direction. In order to facilitate analysis, the stress states at the times of *t* = 20 s, *t* = 40 s, *t* = 60 s, *t* = 80 s, and *t* = 100 s are chosen to refer to the whole corner filling process. In Figure 12, it is found that point M is subjected to tensile stresses in the hoop and axis direction and a compressive stress in the thickness direction at the beginning of the corner filling, but as the corner gets smaller and the tube contacts the die better, the tensile stresses turn into compressive stresses and then point M is subjected to a three-compressive stress state. As a result, the deformation of the middle area of the straight wall mainly occurs at the beginning of corner filling. However, point T is always subjected to tensile stresses in the hoop and axis direction and a compressive stress in the thickness direction. It is conducive to deformation and the maximum effective strain and strain rate all occur at the transition zone. Point C_i_ is subjected to three compressive stresses, while point C_o_ is subjected to tensile stresses in the hoop and axis direction, which denotes a plane-stress state. As a result, the outside of the corner can deform more easily than the inside of the corner and the strain is very different along the thickness direction.

The mean stresses at the typical feature points during the corner filling process are also shown in Figure 12. For point M, the algebraic value of the mean stress changes from a positive number to a negative number and then decreases gradually. This indicates that the tensile stress in the hoop direction is large at the beginning of the corner filling, but it decreases and turns into compressive stress as the tube contacts the die better. Then the hydrostatic pressure increases gradually to a constant value of 30 MPa due to the corner filling process at 30 MPa. But the middle area of the straight wall can hardly deform due to the constraints of the die and the material around. For point T, although the stress state of two-tensional and one-compressive makes the transition zone deform easily, the mean stress decreases gradually during the corner filling process. As a result, the corner filling rate decreases gradually at constant pressure levels. The strain types are both plane strain type at points C_i_ and C_o_, but the loading locus of the mean stress at point C_i_ is very different from that at point C_o_. The mean stress is always positive at point C_o_ and negative at point C_i_. This also indicates that the outside of the corner can deform more easily than the inside of the corner during the corner filling process. The absolute values of mean stresses at points C_i_ and C_o_ both decrease gradually during the corner filling process, which are similar to point T. During the corner filling process, although their strain types are all plane strain type, different areas of the cross-section are subjected to different stress states and this results in different strain and strain rates.

### 3.5. Microstructure Evolution during the Corner Filling Process

**Figure 13:**
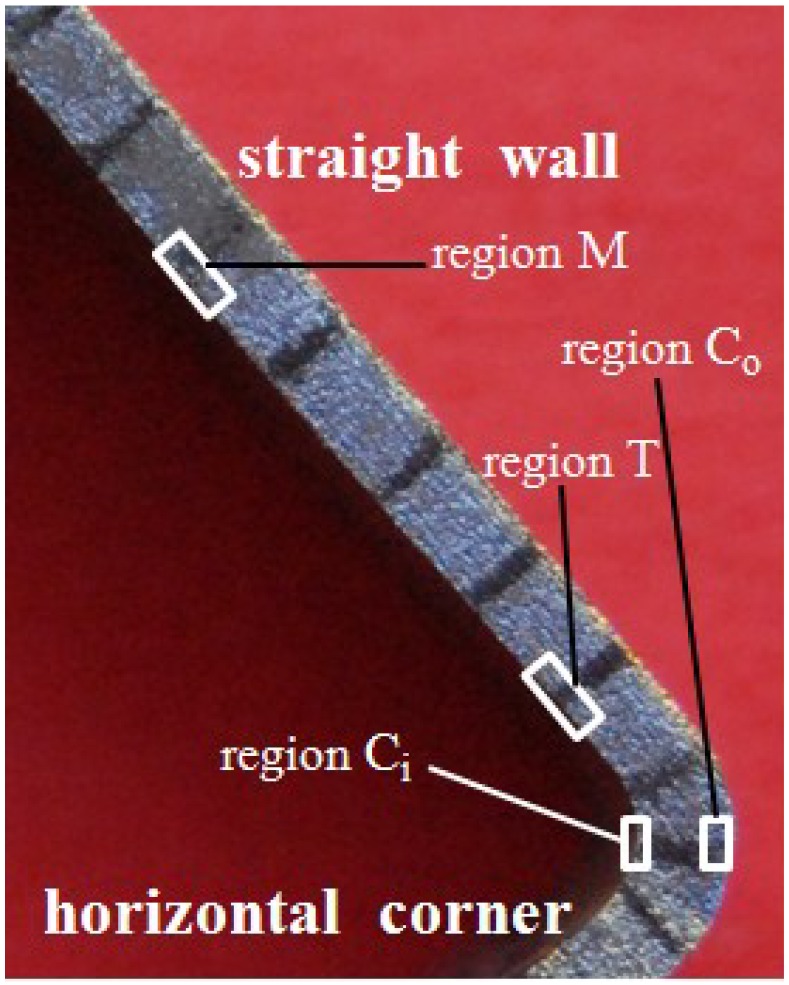
Regions for electron back scattering diffraction (EBSD) analysis.

**Figure 14:**
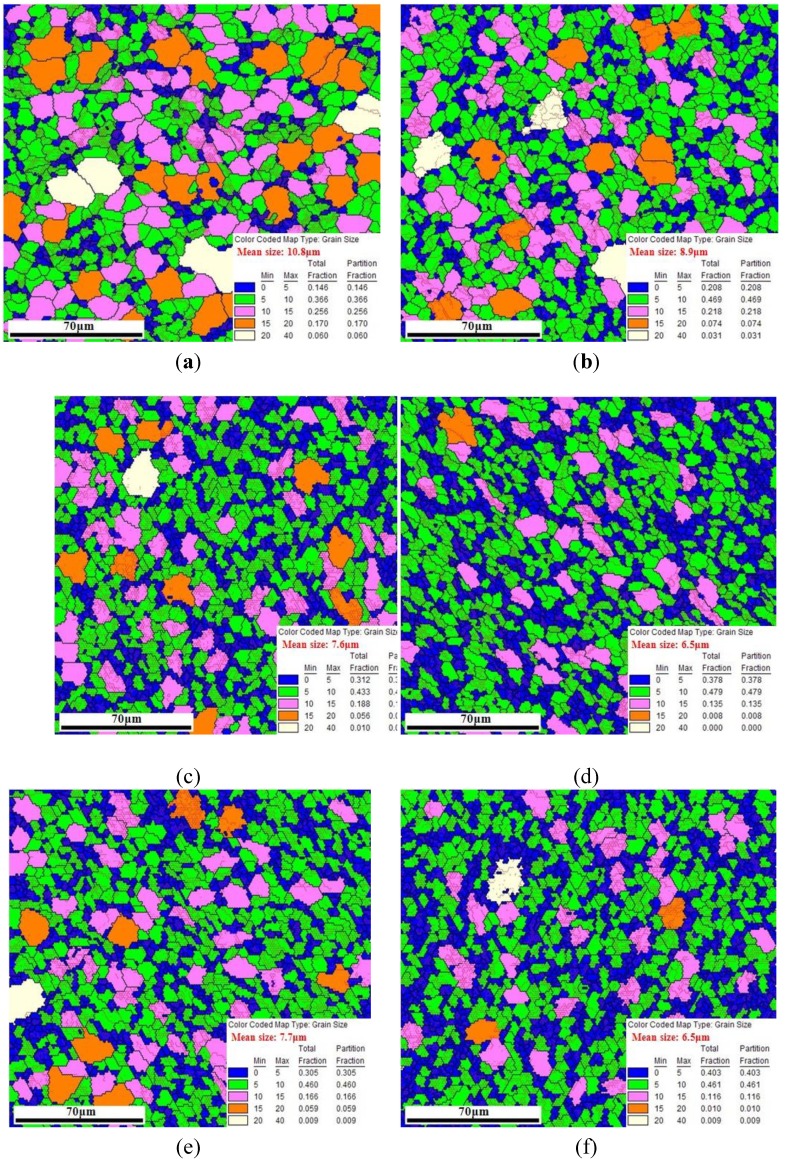
(**a**) Cross-sectional EBSD result of as-received tube; (**b**) cross-sectional EBSD result at region T formed at 850°C and 15MPa; (**c**) cross-sectional EBSD result at region M formed at 850 °C and 30 MPa; (**d**) cross-sectional EBSD result at region T formed at 850 °C and 30 MPa; (**e**) cross-sectional EBSD result at region C_i_ formed at 850 °C and 30 MPa; (**f**) cross-sectional EBSD result at region C_o_ formed at 850 °C and 30 MPa.

From the results of FEM simulation above, it was evident that different regions at the cross-section of the tube are subjected to different stress states during the corner filling process and this results in different strains, so the microstructure at typical regions formed at 850 °C and 30 MPa were analyzed using EBSD. As for the FEM analysis, the areas between the middle of the straight wall area and the horizontal corner area were chosen to be analyzed. Besides, the same region formed at 850 °C and different pressure levels was also analyzed to investigate the effect of forming time on the microstructure. As shown in Figure 13, regions M, T, C_i_ and C_o_ are typical regions and refer to the middle region at the inside of straight wall area, the region at the inside of the transition zone, the central region at the inside of the corner and the central region at the outside of the corner respectively. Figure 14a depicts the EBSD result showing the microstructure of the as-received tube. The initial grain size in the scanned area varies over a wide range with the mean size of 10.8 μm. The microstructures of typical regions formed at 850 °C and 30 MPa are shown in Figure 14c–f: overall, the grains of four regions are all refined to a certain extent, but the refining effect is more obvious with larger deformation; the grains are refined with mean size of 6.5 μm at regions T and C_o_, and the grains are refined with mean size of 7.6 and 7.7 μm at regions M and C_i_ respectively. As shown in Figure 9, the effective strain is larger and similar at regions T and C_o_, and it is smaller and similar at regions M and C_i_, thus the results above occur. For regions T and C_o_, the proportion of the grains with size smaller than 5 μm is 37.8% and 40.3% respectively, but for regions M and C_i_, it is 31.2% and 30.5% respectively. It is probably because a larger deformation can strengthen the dynamic recrystallization at high temperature. As shown in Figure 14b, the grains are refined with mean size of 8.9 μm at region T formed at 850 °C and 15 MPa. Compared with region T formed at 850 °C and 30 MPa, the refining effect is less significant. It is also found that the proportion of the grains with size smaller than 5 μm is 37.8% at 30 MPa but only 20.8% at 15 MPa. This is probably because some fine grains after dynamic recrystallization grow due to the much longer forming time at 15 MPa. The results illustrate that the HPPF at high pressure has advantages both for efficiency and microstructure. Although the grain size of the formed components at high pressure is not as uniform as the original tube blank, the difference is acceptable.

## 4. Conclusions

In the presented work, high pressure pneumatic forming of Ti-3Al-2.5V titanium tubes in a square cross-sectional die was investigated from the aspects of experimental study, numerical simulation, and microstructure analysis. Conclusions can be drawn from this work as follows:

Because of the elevated temperature and high pressure levels in the HPPF process, the deformation behavior of Ti alloy is affected both by work hardening effect and strain rate sensitivity. The corner radii of the forming tubes depend on the internal pressure levels and forming time, and the higher pressure level can significantly reduce the forming time for runs at the same temperature. For the corner filling process at constant pressure levels, the strain rate decreases gradually as the corner radius reduces, but it does not drop to zero due to the effect of strain rate sensitivity at elevated temperature.

Because of different stress states during the corner filling process, the strain and strain rate are very different at different areas of the tube. The maximum strain and strain rate both occur at the transition zone, because this area is always subjected to two-tensional and one-compressive stress state. The straight wall area is subjected to two-tensional and one-compressive stress state at the beginning of corner filling, but is subjected to a three-compressive stress state as the corner gets smaller. Small deformation at the straight wall area mainly occurs at the beginning of corner filling. For the corner areas, the strain is very different along the thickness direction, because the inside of the corner is subjected to three-compressive stress state while the outside of the corner is subjected to two-tensional stress state. Overall, through the HPPF process, the maximum thinning ratio of the cross section is smaller than 15%, which can meet the demand of most applications, although the stress states are different at different areas.

The grains can be refined to a certain extent after the HPPF process in a square cross-sectional die. Because of different deformation caused by different stress states, refining effects at the transition zone and outside of the corner are more obvious than those at the straight wall area and inside of the corner. The longer forming time at the lower pressure level can also make the refining effect less significant due to fine grains growing at high temperature. Although the grain size of the formed components at high pressure is not as uniform as the original tube blank due to different stress states at different areas, the difference is acceptable, and a better microstructure can be obtained at a higher pressure level for forming at elevated temperature.
